# Evolutionary, Comparative and Functional Analyses of the Brassinosteroid Receptor Gene, *BRI1*, in Wheat and Its Relation to Other Plant Genomes

**DOI:** 10.1371/journal.pone.0127544

**Published:** 2015-05-28

**Authors:** Christopher Navarro, Jerott Moore, Alina Ott, Eric Baumert, Amita Mohan, Kulvinder S. Gill, Devinder Sandhu

**Affiliations:** 1 Department of Biology, University of Wisconsin-Stevens Point, Stevens Point, Wisconsin 54481, United States of America; 2 Department of Crop & Soil Sciences, Washington State University, Pullman, Washington 99164, United States of America; University of Missouri, UNITED STATES

## Abstract

Brassinosteroids (BRs) are plant hormones, fundamental for the growth and development of plants. A trans-membrane protein receptor kinase, Brassinosteroid-Insensitive 1 (BRI1), is known to interact with BRs and be directly involved in plant development. This study investigates the structural organization of *BRI1* orthologs in several taxa, with a specific interest in *Triticum aestivum*. True orthologs of *Arabidopsis thaliana BRI1* (*AtBRI1*) from seven-plant species showed sequence identity ranging from 54% to 95% at the protein level. All gene sequences lacked introns, leading to speculation that post-transcriptional processing in *TaBRI1* is similar to *AtBRI1*. Based on *in silico* analysis, a single copy of *BRI1* was present in each of the three wheat genomes on the long arm of chromosome 3. Domain structure of BRI1 orthologs among different taxa showed multiple leucine rich repeats (LRRs), an island domain (ID), a juxtamembrane/transmembrane domain (JTMD), a catalytic kinase domain (KD), C and N-Terminal domains. The KD showed the highest level of conservation while the LRRs and JTMD were most variable. Phosphorylation of residues in the juxtamembrane domain, known to be involved in the activation of the KD, is conserved in TaBRI1. While TaBRI1 has well-defined differences in the ID and LRR domains, many residues involved in ligand binding are conserved. The activation loop present in the KD showed 100% conservation in all taxa. Despite residue differences, hydrophobicity was conserved in the BR binding pocket across taxa, suggesting that function may not differ as drastically as residue identity may suggest. Predicted 3D structure of AtBRI1 and TaBRI1 showed a conserved super helical assembly, a feature essential in protein-protein interactions. An unrooted phylogram showed TaBRI1 in the monocot clade to be distinct from that of dicots. New insight in the structure and functions of BRI1 may help in targeting BR pathway for crop improvement.

## Introduction

Steroid hormones play important role in the growth and development of living organisms. Brassinosteroids (BRs) are known to be involved in various physiological responses including: cell division, cell differentiation, stem elongation, male fertility, vascular development, flowering, photomorphogenesis and responses to environmental stresses [1–4]. BR is perceived at the cell surface by the BRI1 protein, a transmembrane hormone receptor [5,6]. Signal cascades involving BRI1 regulate developmental processes and are known to modulate the gibberellin pathway as well [7–9]. By measuring fluorescence resonance energy transfer between BRI1-CFP and BRI1-YFP, it is known that BRI1 exists in the plasma membrane as a homo-oligomer [10]. Its interactions in the plasma membrane are also dependent on the presence of leucine rich repeats (LRRs), domain structures that are essential in protein-protein interactions. While many of the components of the BR signaling pathway have been identified and characterized, BRI1 and its mechanisms in crop plants are under-explored.

The signaling cascade of BRI1 is understood in Arabidopsis. The presence BRI1 in the plasma membrane as a homo-oligomer is known to be BR independent, a feature that brings into question how kinase activity is dependent on other proteins [11]. BRI1 Kinase Inhibitor 1 (BKI1) has been identified as a substrate of BRI1 kinase and acts as a negative regulator in the BR-mediated pathway [12,13]. Dependent on BR, BKI1 is released from the BRI1 homo-oligomer, allowing other BRI1 associated proteins to associate with BRI1 [13]. Autophosphorylation in some plant kinases, including BRI1, has been known to occur in Ser/Thr and Tyr residues, identifying BRI1 as a dual specificity kinase [14]. At high levels of BR, activated BRI1 can interact with its counterpart, BRI1 Associated Receptor Kinase 1, otherwise known as Somatic Embryogenesis Receptor-Like Kinase (SERK3), and form a BRI1/SERK3 complex known to undergo transphosphorylation [15]. SERK3 is therefore known to be involved in BR signaling and its under-expression induces a dwarfed phenotype, as seen in the BRI1 mutant [12,15].

Subsequent steps after the formation of the BRI1/SERK3 heterodimer involve phosphorylation of Brassinosteroid-Signaling Kinases (BSKs), which represent a small family of proteins that induce downstream signaling of BRI1 [16]. BSK’s substrate, Brassinosteroid Insensitive 2 (BIN2), is known to be a negative regulator of the BRI1 pathway [17,18]. In the absence of BRs, BIN2 phosphorylates two transcription factors, Brassinazole Resistant 1 (BZR1) and BRI1-EMS-Suppressor 1 (BES1)/BZR2, rendering them inactive [16,19]. In the absence of BIN2, BZR1 and BES1/BZR2 are dephosphorylated by Protein Phosphatase 2A (PP2A) [20]. The dephosphorylated active transcription factors, BZR1 and BES1/BZR2, can then bind to specific sites on DNA and regulate expression of several genes [21,22].

The *BRI1* gene shares structural similarity to a gene family of receptor-like kinases (RLKs). RLKs have at least 600 members that represent nearly 2.5% of Arabidopsis protein coding genes [23]. The BRI1 protein contains LRR motifs, an island domain (ID), a single pass juxtamembrane/transmembrane domain (JTMD), a kinase domain (KD), a C-terminal cap (CT) and an N-terminal cap (NT) [6]. LRRs in the extracellular domain of BRI1 are known to be involved in protein-protein interactions, specifically heterodimerization with SERK3. [11,24]. There is also an ID sandwiched among the LRRs that contributes to ligand binding of BRI1. Brassinolide (BL), a commonly found BR, is a ligand of BRI1 and interacts with this 70 residue long ID between LRRs 21 and 22 [5]. BRI1 does afford some accommodation to its ligand, and has been known to bind with several BRs similar to BL [25]. This brings into question the structural basis of BR binding within the ID, a topic that will be investigated further in this study.

Orthologs of *BRI1* are ubiquitous in the plant kingdom and BR sensitivity in the *BRI1* orthologs has been studied in tomato, pea, rice and barley [26–30]. The *BRI1* loss of function mutant in *Oryza sativa* showed significant reduction in height with little effect on fertility, making *BRI1* an ideal candidate for alternatives to gibberellins-affected dwarfs [31]. The *BRI1* mutant in *Hordeum vulgare* showed a phenotype with characteristics similar to other BR insensitive mutants [28]. The focus of this study is to look at the BRI1 protein in the context of structural and functional features across several taxa with an emphasis on wheat. A cross-species analysis of BRI1 gives a foundation for investigating BRI1 in crop plants where BRI1 is not well studied. Because BRI1 has such an effect on the meristem development, it is likely that this gene can be silenced in wheat to affect phenotype, specifically height. By using comparative analysis, an overview of the protein structure and predicted function of TaBRI1 in the context of other taxa can be made.

## Materials and Methods

### Gene Sequence Retrieval

Using *Arabidopsis thaliana BRI1* (*AtBRI1*) as a query sequence, cDNA sequences for single copies of *BRI1* for *Triticum aestivium* (wheat, DQ655711); *Sorghum bicolor* (sorghum, Sb03g032990); *H*. *vulgare* (barley, AB109215.1); *Zea mays* (corn: GRMZM2G048294); *O*. *sativa* (rice, LOC_Os01g52050); *Brachypodium distachyon* (Brachypodium, Bradi2g48280); and *Glycine max* (soybean, Glyma04g39610) were retrieved from NCBI and Phytozome (http://www.ncbi.nlm.nih.gov; http://www.phytozome.net/).

### In-silico physical localization of *TaBRI1*

The *TaBRI1* gene sequence was used to physically localize the gene on wheat chromosome using the mapped wheat EST database available at GrainGenes (http://wheat.pw.usda.gov/GG3/blast) using the default parameters. Further, the chromosome specific wheat survey sequence data available by IWGSC was accessed through ViroBLAST [32]. *TaBRI1* was used as a query sequence to BLAST against all the available chromosomes (https://urgi.versailles.inra.fr/blast/blast.php) using the default parameters. BLAST hits with an E-value 0 were considered for mapping.

### *In-silico* expression analysis of *TaBRI1*

Wheat 61K microarray data was assessed via the PLEXdb portal (www.plexdb.org/modules/tools/plexdb_blast.php) to perform the *in-silico* expression of the *TaBRI1* gene [33].

### Conserved Domain Prediction and Sequence Comparisons

Predicted amino acid sequences of all the species were aligned to generate a consensus sequence using the ClustalW software with barley as a reference. The domains and motifs annotation of the consensus sequence was predicted by Conserved Domain Database (CDD) (http://www.ncbi.nlm.nih.gov/Structure/cdd/wrpsb.cgi). In addition, the phasing and location of LRRs were determined by entering sequences into PFAM and predicting secondary structure [34]. Similarity plots were drawn for dicots and monocots separately. All the 6 monocots and 2 dicots were then compared with the resulting consensus sequence to draw sequence similarity at each position of protein. The scale of 6 for monocots indicates amino acid similarity at each particular position in all 6 monocots whereas 1 indicates residue presence in 1 species only. Similarly for dicots, a scale from 0 to 2 was assigned, where 2 indicated presence in both species and 0 indicates the absence conservation of amino acid in dicots compared to consensus sequence.

### Prediction of 3-D structure

Predicted BRI1 protein sequences from all species were used to model the 3-D structure using PHYRE^2^—Protein HomologY Recognition Engine (www.sbg.bio.ic.ac.uk/phyre2/) and RaptorX (Template-based protein structure modeling using the RaptorX web server) [35]. The search engine is based on the Structural Classification of Proteins database and augmented with the Protein Data Bank for remote homology detection.

### Phylogenetic Analysis

Protein sequences were aligned with ClustalW (http://www.ebi.ac.uk/Tools/msa/clustalw2/). The phylogenetic phylogram of all aligned sequences was constructed using the MEGA 5.05 software [36]. The evolutionary distances were computed using the Poisson correction method and are in units of the number of amino acid substitutions per site. The evolutionary relationship was inferred by Neighbor-Joining method of distant matrix. Despite a small sample size, bootstrap re-sampling with 1000 replicates was used to provide support for all groups (>95%).

## Results

**Fig 1:**
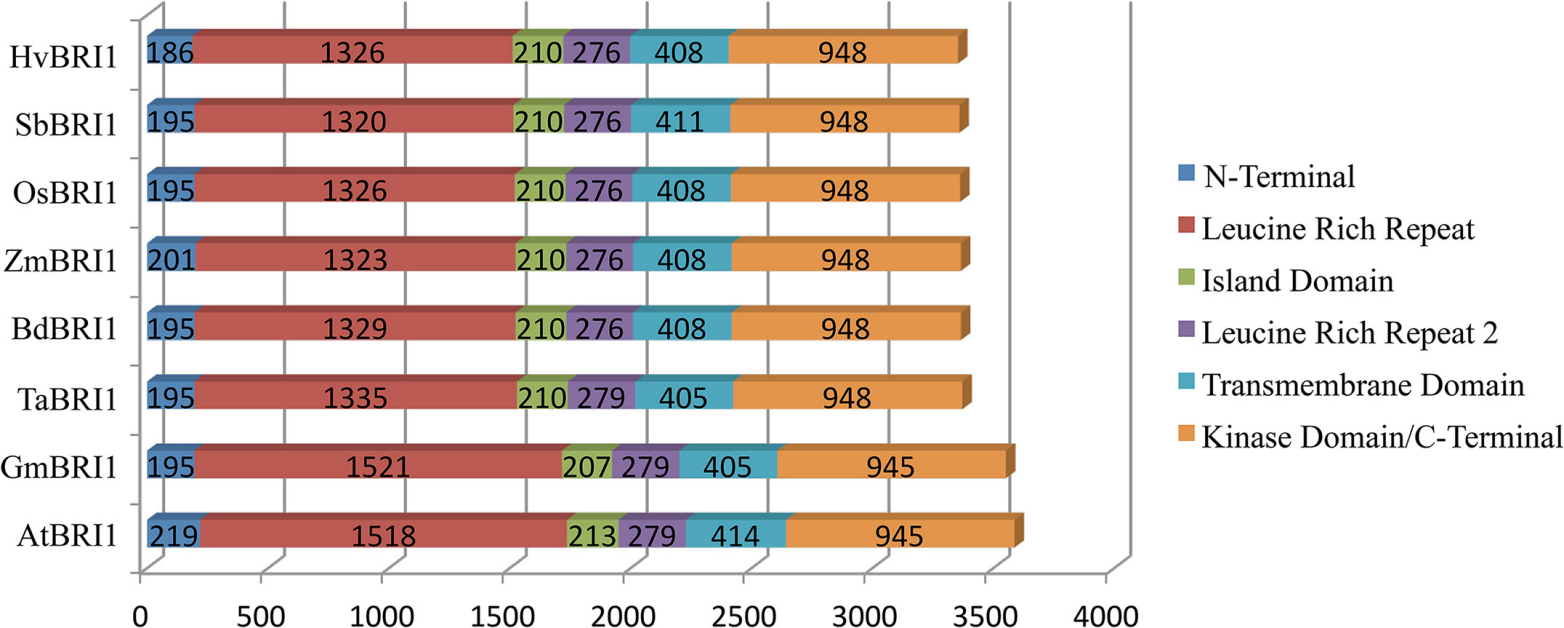
Schematic representation of domain lengths for *BRI1* in two dicot and six monocot species. Nucleotide length in base pairs is shown for each domain. All full sequence genes lacked introns. A difference in length between monocots and dicots can be observed.

*TaBRI1* has high nucleotide sequence identity with *HvBRI1* (94%), *BdBRI1* (89%), *OsBRI1* (84%), *SbBRI1* (82%), *ZmBRI1* (82%), *AtBRI1* (74%) and *GmBRI1* (69%) (Table 1). Manual assembly of contigs for three wheat genomes revealed the absence of introns, consistent with the gene structure of *TaBRI1*’s orthologs (Fig 1). Gene lengths for all taxa analyzed ranged from 3,354 to 3,588 bp (Fig 1). The domains that showed significant variation in length were the LRRs, which ranged from 1,320 to 1,335 bp in monocots and from 1,518 to 1,521 bp in dicots. Overall, dicot gene sequences were longer than monocot gene sequences. Protein comparison of TaBRI1 with 97% coverage showed 95% identity with HvBRI1, 90% with BdBRI1, 83% with OsBRI1, 80% with SbBRI1, 79% with ZmBRI1, 54% with AtBRI1 and 56% with GmBRI1 (Table 1).

**Table 1:** Percent identity comparison of the *TaBRI1* gene and the TaBRI1 protein with five monocot and two dicot species.

	*H*. *vulgare*	*B*. *distachyon*	*O*. *sativa*	*S*. *bicolor*	*Z*. *mays*	*A*. *thaliana*	*G*. *max*
Gene	94%	89%	84%	82%	82%	74%	69%
Protein	95%	90%	83%	80%	79%	56%	54%

The ortholog for *AtBRI1* in *T*. *aestivum* were physically localized on chromosome 3 using the available information at GrainGenes. The wheat survey sequence for the individual chromosome and chromosome arms further confirms its localization on long arm of homeologous group 3. Significant blast hits on 3AL, 3B and 3DL suggested that at least three homeologous copies of *TaBRI1* are present in bread wheat, an expected result due to hexaploid nature of the wheat genome. Chromosome 3B sequence showed 99% identity at nucleotide level for the complete length of the gene. However, a wheat EST mapped to deletion bin C-3AL3-0.42 on chromosome 3AL also showed 99% identity with the *TaBRI1*. Due to unavailability of complete sequences for three wheat genomes at this time, it is not possible to determine which copy matches closely with *AtBRI1*.

**Fig 2:**
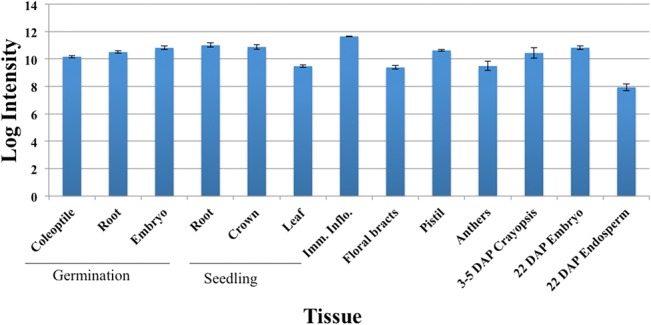
*In-silico* expression analysis of the *TaBRI1* gene using the wheat 61K Affymetrix GeneChip data. Error bars represent standard errors. Three independent biological samples represented a tissue type.

The *in-silico* expression analysis of *TaBRI1* using the transcription pattern during wheat development identified two probes with 95.6% and 89.7% identities. Probe set (Ta.9744.1.S1) showing 95.6% identity with an e-value of 0.0 was used to predict the gene expression pattern. The gene was expressed in all the developmental stages ranging from early germination to differential developmental stages of flower (Fig 2). The highest expression was observed in immature inflorescence with a lowest in 22 days after pollination (DAP) embryo (Fig 2). *TaBRI1* showed high expression in the coleoptile and the crown regions, however, due to unavailability of expression data at the ‘jointing stage’ made it impossible to establish any relationship between *TaBRI1* expression and plant height.

**Fig 3:**
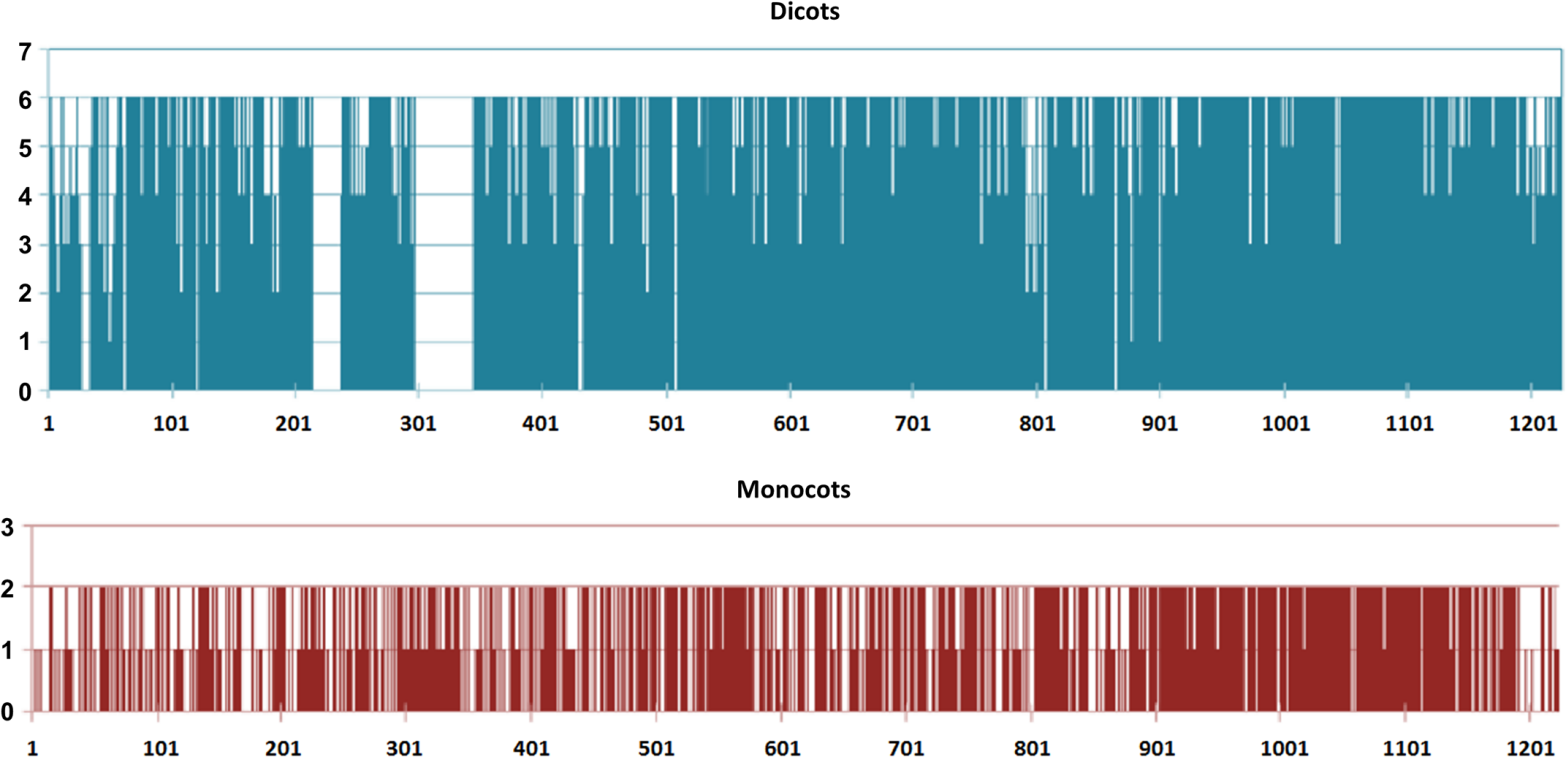
Conservation of amino acid residues of BRI1 proteins in monocots (blue) and dicots (red). Conservation is represented in the form of bars showing a 0 to 6 level of conservation in monocots or 0 to 2 level of conservation in dicots for each residue. Regions found only in the dicot sequences are represented with a score of 0 in the monocot conservation graph and vice versa.

**Fig 4:**
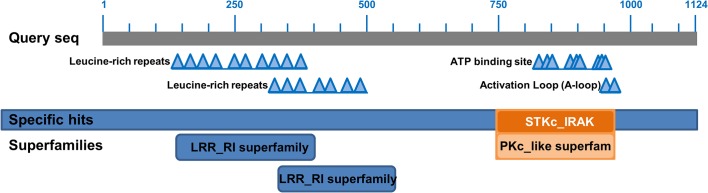
Representation of different domains present in the TaBRI1 protein. Conserved domains are represented along the sequence. LRRs were detected in the beginning of the sequence and the inter-cellular domain consisted of ATP binding sites and an activation loop. A protein kinase c (PKc) superfamily was detected which reaffirmed BRI1’s catalytic ability.

Sequences of six monocots and two dicots were compared in similarity plots to study the conservation of the protein across monocots (red) and dicots (blue) (Fig 3). The highest levels of conservation were seen in the KD, ranging from residues 900 to 1200 (Fig 3). Three regions present in dicots but missing in monocot sequences were of interest and could be found between residues 26–32, 199–220 and 280–326. There were few regions where residues present in monocots were missing in dicots, but none were longer than three residues (Fig 3). The TaBRI1 sequence was then searched in the CDD (Fig 4) (http://www.ncbi.nlm.nih.gov/Structure/cdd/cdd.shtml) [37]. Multiple LRRs were detected in the CDD from residues 60 to 750 (Fig 4). A section ranging from 580 to 700 contains no LRRs and is presumably the ID. A catalytic protein kinase (PKc) domain was detected from residues 900 to 1200 (Fig 4). ATP binding sites along with an activation loop were also detected within the KD.

**Fig 5:**
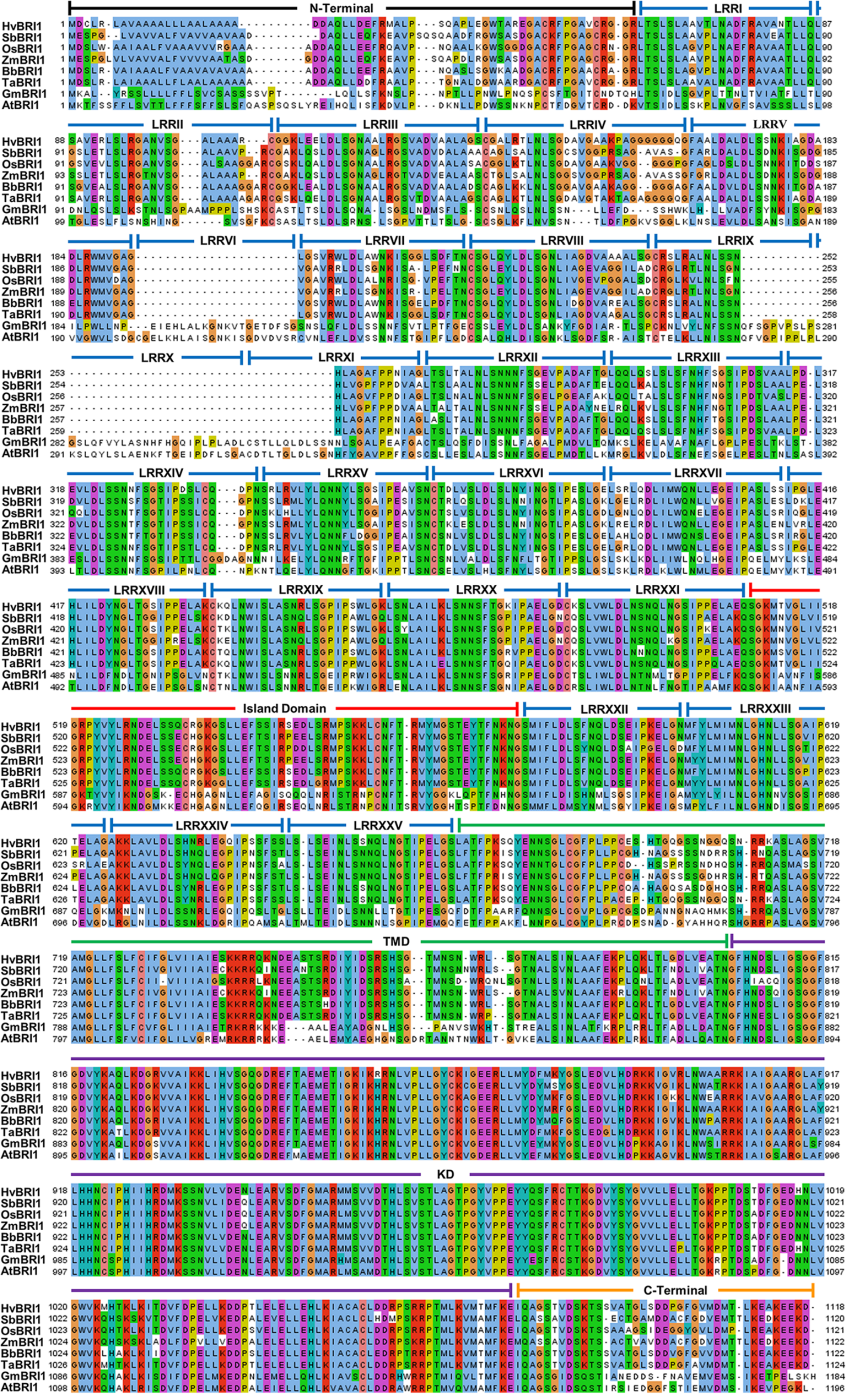
Protein comparison of BRI1 sequences in eight plant genomes. The alignment comparison was performed by ClustalW. Colors represent the type of residues with solid colored residue columns representing conservation. Domains are represented by horizontal bars on top of the sequences.

Comparisons of all six monocot and two dicot BRI1 sequences revealed that all 25 LRRs were found in the dicot sequences, but monocots lacked LRRs VI and X, and showed partial sequence conservation for LRRs IX and XI (Fig 5). The number of LRRs before and after the ID was defined as N_1_ and N_2_, respectively. The ratio N_1_/ N_2_ was 4.94 and 4.50 for dicots and monocots, respectively. The highly conserved LRR sequence among all taxa was found to be LxxLxLxxN followed by an Asn or polar residue and highly conserved Cys (Fig 5).

**Fig 6:**
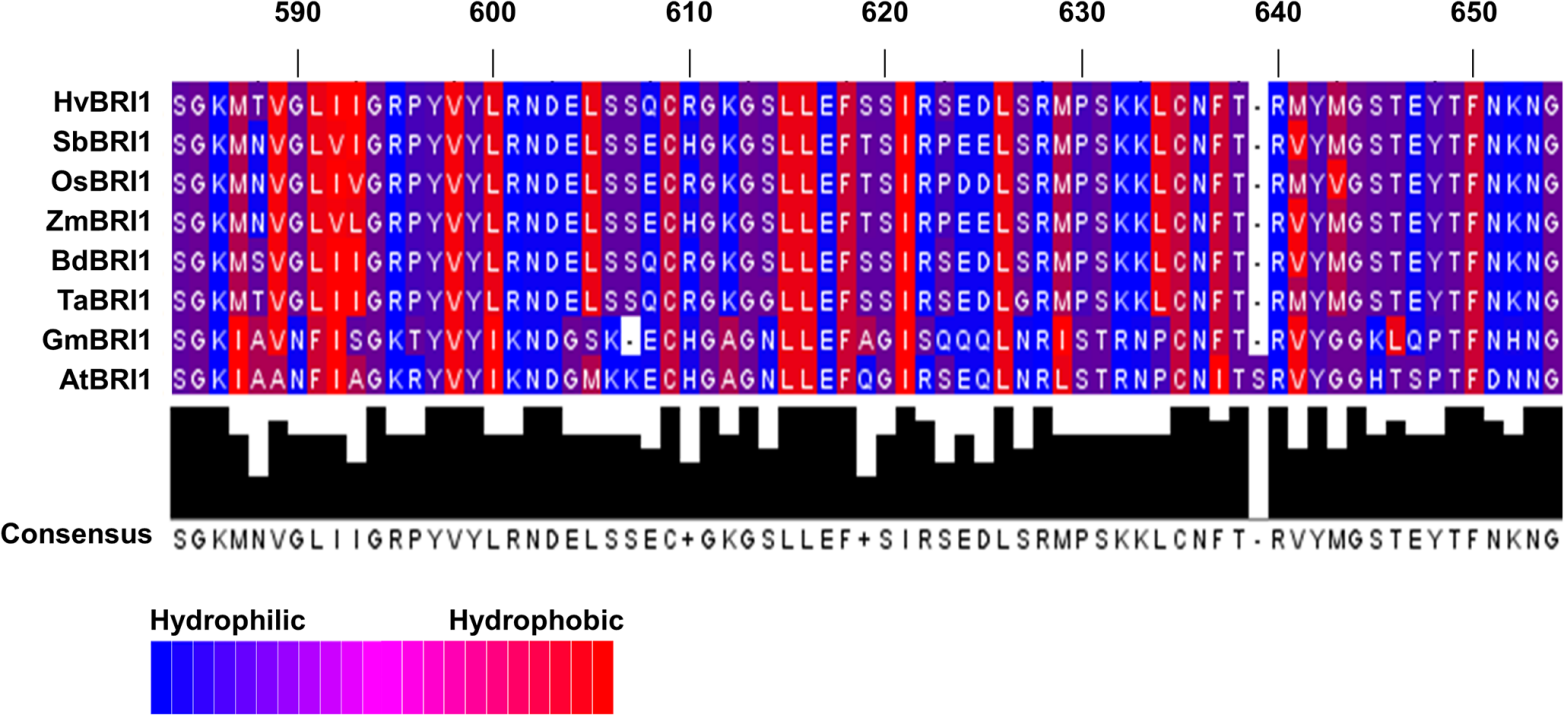
Comparisons of island domain (ID) of BRI1 in different plant species. The ID ranged from residues 580 to 649 with respect to AtBRI1. The residues are colored based on their hydrophobicity or hydrophilicity. Significant differences can be seen in the two different clades of plants, with dicots showing more hydrophobic regions. There is also variation in the total length of the ID: dicots have 69–70 residues, while monocots have 68 residues.

Detailed analysis of ID revealed an overall high conservation of hydrophobic residues, but notable differences between dicot and monocot sequences could be observed (Fig 6). Wheat and other monocot sequences had a conserved L-605 residue that was substituted instead with more hydrophilic Met and Ser residues in AtBRI1 and GmBRI1, respectively (Fig 6). Dicots also lacked L-634, a conserved hydrophobic residue in monocots (Fig 6). The three-residue motif found in F-591, I-592 and A-593 region in AtBRI1 was more hydrophilic in the dicot sequences and was substituted with the slightly more hydrophobic Leu or Ile residues in monocots (Fig 6).

The KD and JTMD possessed the greatest level of conservation across all taxa. T-842, known to be a KD modulating site [38], was conserved in all taxa except GmBRI1 (Fig 5). S-887 was conserved in all taxa except for OsBRI1, which had a Cys residue. T-872, T-880, Y-956, S-891, S-858, and Y-831, all important phosphorylation sites [39], were conserved in all sequences. T-1049, T-1045, S-1042, S-1044, T-1039 and S-1060, also important residues in the activation loop [39], were conserved in all sequences (Fig 5). T-982, which is also phosphorylated in AtBRI1 activation loop [38], was only found in SbBRI1 and in other sequences was substituted with Ala or Ile residues (Fig 5).

**Fig 7:**
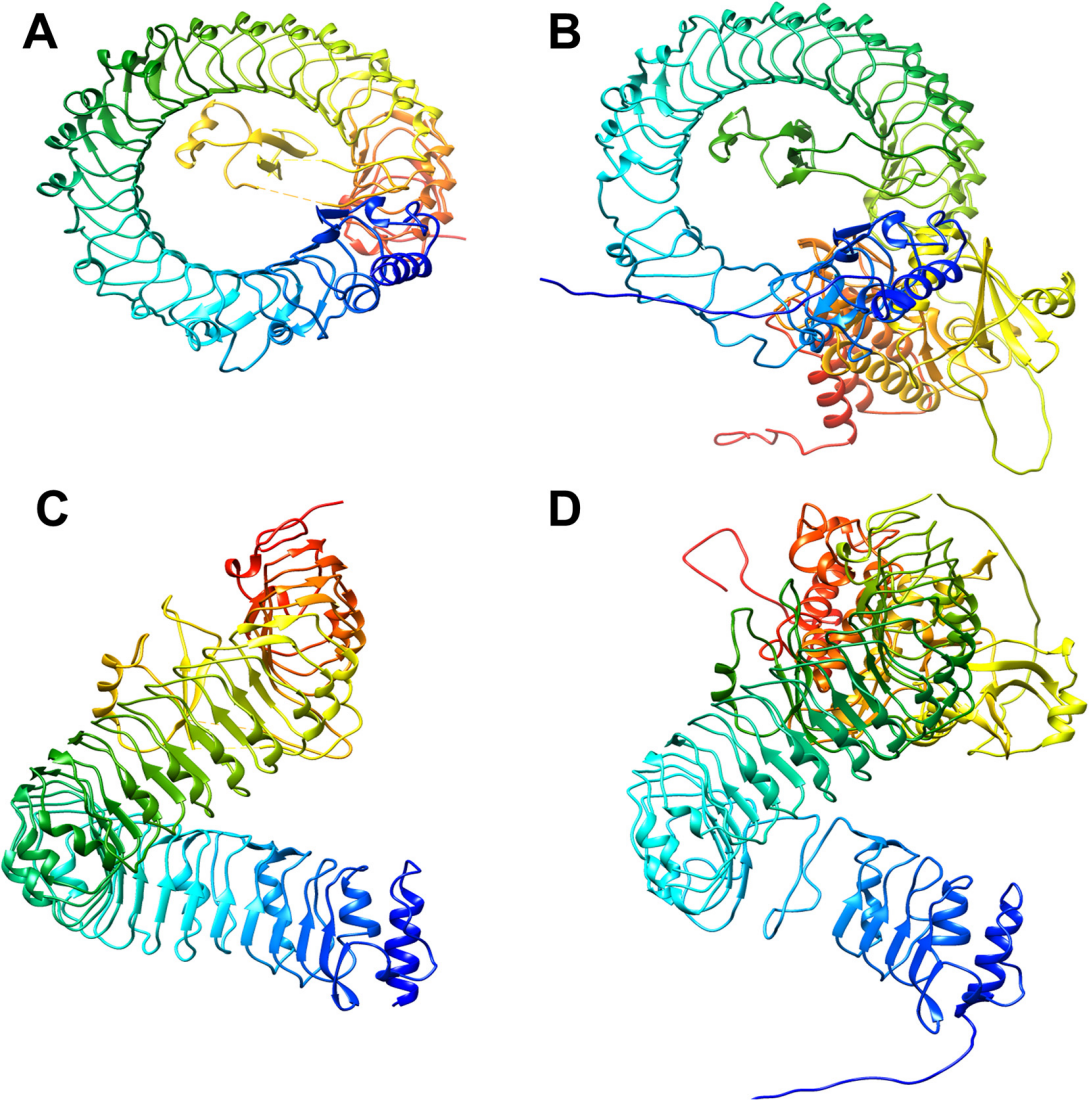
Comparison of predicted 3D structures of AtBRI1 and TaBRI1 proteins. A, B: AtBRI1 protein; C, D: TaBRI1 protein. A conserved LRR can be observed in the large helical structure shown in blue and green. Yellow and red colors likely represent the KD, with the red region representing the substrate binding site.

When compared to the structure of the characterized AtBRI1, the predicted protein structure of TaBRI1 is remarkably similar (Fig 7). Both proteins contain an extracellular LRR region that results in the formation of a large helix comprised of alpha helices and beta pleated sheets (Fig 7). Where most LRR motifs result in a horseshoe structure, the LRR in AtBRI1 forms an uncommon super helix. The fact that the estimated protein structure of TaBRI1 also contains this super helix is in itself compelling evidence for functional similarity. The super helix region is structurally unaffected by BL binding, but it is believed that it plays a key role in the activation of the BR perception pathway via protein-protein interactions [6]. Within the concave inner circle of the helix lies the hydrophobic insertion domain where BL binds [6]. The predicted protein structure of TaBRI1 shows similarity in the proximity of the conserved insertion domain when compared to the known structure of AtBRI1 (Fig 7). The insertion domain in BL bound AtBRI1 has been shown to have marked structural differences when compared to free AtBRI1. This change in structure is suspected to contribute to the perception of BL and the activation of the BRI1 pathway.

**Fig 8:**
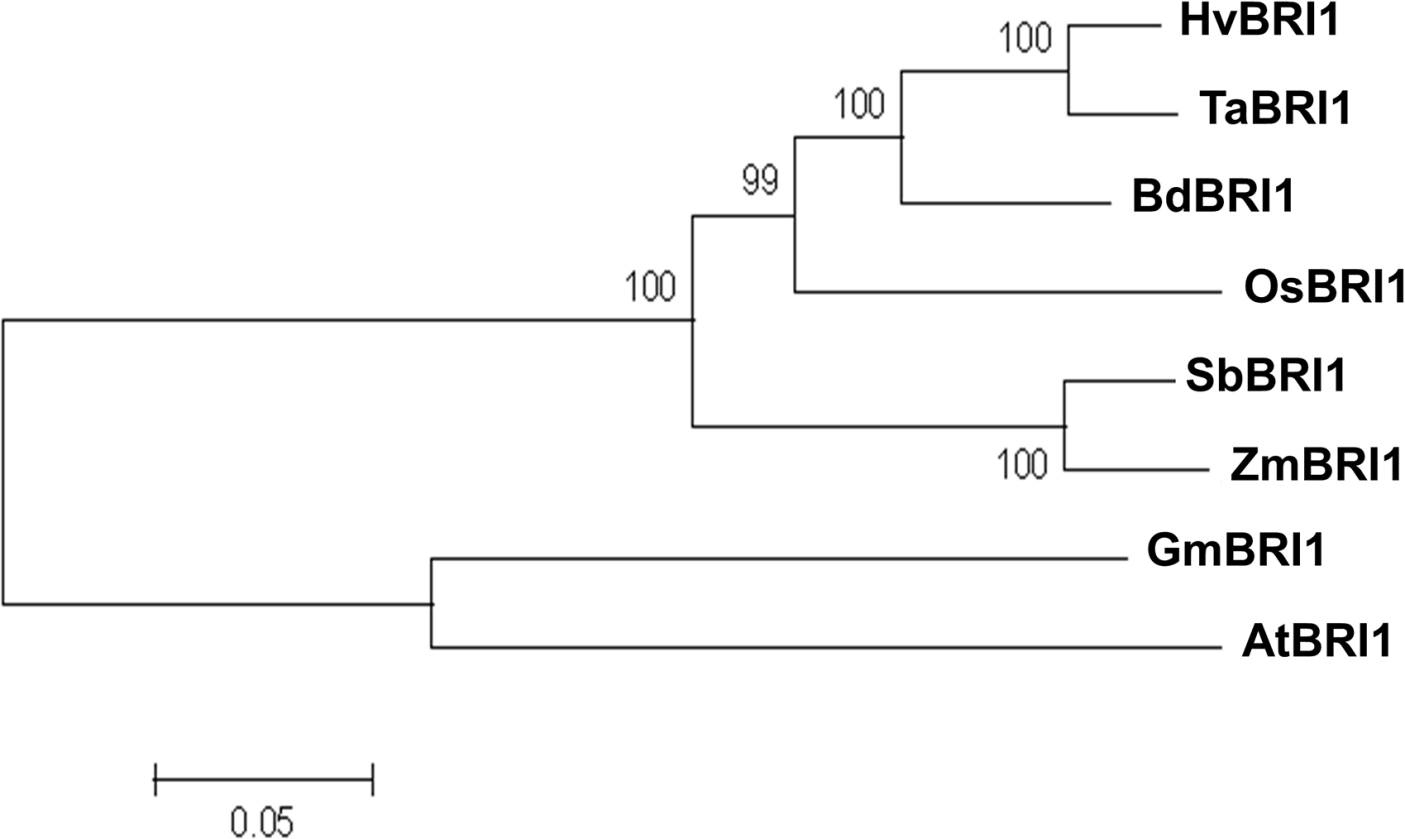
Phylogenetic analysis of TaBRI1 and its orthologs from the seven examined plant species. Two distinct clades can be observed consisting of monocot and dicot sequences. As expected, TaBRI is most closely conserved with HvBRI1.

The total sequence alignment was used to compute an unrooted phylogram showing evolutionary divergence of TaBRI1 and 7 other primary sequences (Fig 8). The rectangular phylogram has external nodes representing each protein sequence and internal nodes with percentage support for each divergence. Branch distance is shown in units of residue substitutions per site. Supported by the sequence differences between monocots and dicots, two different clades are formed in the phylogenetic tree of all taxa (Fig 8). Furthermore, additional BRI1-like genes from taxa of both the dicot and monocot genomes are displayed in a phylogeny supporting this difference (S1 Fig). Dicots had a clade of their own, distinct from the monocot sequences. While GmBRI1 and AtBRI1 were in a group of their own, their node branch lengths show relatively long divergence (Fig 8). In monocots, TaBRI1’s closest relative was HvBRI1. BdBRI1 placed close to TaBRI1 and HvBRI1, a likely outcome as *Brachypodium diastachyon* is closely related to the tribe Triticeae (Fig 8)) [40]. OsBRI1 shared close similarity to BdBRI1, but was not as close in similarity to ZmBRI1 and SbBRI1. These observations are in line with the evolutionary history of these taxa.

## Discussion

### BRI1 is highly conserved across taxa

Plant RLKs are known to be involved in diverse biological roles that include hormone response, plant growth and stress response [41]. Because of their diverse functions, a wide variation in domain structure of gene families can be observed. For BRI1, however, a high level of similarity can be observed in different taxa. The conservation seen in the schematic of gene lengths suggests that the relative sizes of BRI1 across species are similar for the two different monocot and dicot groups. The lack of introns in all genes suggests that the mechanism for post-transcriptional processing of *BRI1* is similar in both *A*. *thaliana* and *T*. *aestivum*.

Previous evolutionary studies between rice and Arabidopsis RLKs have shown that the number of RLKs have undergone significant duplication events after their divergence 150 million years ago [42,43]. This evolutionary divergence can be used to explain the difference in LRR ratios seen in monocot and dicot BRI1 sequences and help account for sequence variation seen in the ID and surrounding regions. Furthermore, the variation seen in the Cys residues, essential for disulfide bonding in the LRR domains, may suggest TaBRI1 has co-evolved with its associated proteins.

### Despite differences, LRRs maintain functional similarity in monocots and dicots

Crystal structure of AtBRI1 shows that the LRRs adopt an arc-shaped protein with concave β-strands, convex helices, and a hydrophobic core [44]. The LRR domains of all taxa were identified as groups of 24 residue tandem repeats, known to organize into an arc in the extracellular portion of BRI1 (Fig 5) [45]. Individual LRR domains, which comprise the extracellular domain, correspond to beta-alpha structural units, which arrange to form parallel beta-sheets in a superhelix [28,46]. Differences in numbers of LRRs among species may give insight to how non-LRR IDs occurred in their specific regions of plant RLKs (Fig 5) [47]. While the number of LRR domains between the N-Terminal and the ID possessed variability across taxa, the number of domains between the ID and JTMD was constant across all taxa. In line with this observation, N_1,_ the number of LRRs before the ID, tends to be greater and possesses more variation than N_2_ in plant RLKs [48]. The low N_1_ value in TaBRI1 suggests that BRI1 associated proteins interacting in the extracellular region may have structurally different roles than those seen in AtBRI1. Because LRRs in N_2_ have a purpose in ligand binding, it is possible that LRRs after the ID are more critical for receptor activation, thus their conservation in BRI1 is observed.

Aside from maintaining the hydrophobic core BRI1 solenoid, LRRs play an important role in protein folding and protein-protein interactions [49]. The highly conserved Cys residues in the LRR region are essential for the confirmation of the super-helical assembly [44]. The conserved Cys residues in all the species studied account for disulfide bond formation between LRRs: II to IV, VIII to IX, and XIV to XVI. In AtBRI1 and GmBRI1, a disulfide bond likely forms between LRRs XI and XII [44]. TaBRI1 sequence however, lacks LRRs XI and XII, and likely possesses a condensed super-helical ectodomain due to the shortened LRR domain. C-199 and C-221, known residues in disulfide bond formation, are only present in AtBRI1. Interestingly, GmBRI1 has LRR IV but is completely absent of any disulfide bonding residues but still shows conservation for β-sheet secondary structure assignment [44]. An Asp following the third Leu in the LRR domain was typically found in dicot LRRs but was not seen in LRRs II, IV, VI, X and XI, of the monocot sequences (Fig 5). While this position is also known to be substituted with Cys in plant specific LRRs, no such substitution was observed in any taxa studied [49,50].

The TaBRI1 sequence showed the presence of Ile-Pro spine from LRRs XII to XXV (Fig 7). In Arabidopsis, an Ile-Pro spine was found to run along the outer surface of the protein and provides packing interactions among LRRs IX to XXV [44]. The Ile-Pro spine is found in all taxa and is known to follow a beta strand also involved in LRR packing (Fig 5) [44].

### Similarities in the ID indicate that ligand binding is conserved in different BRI1s

The ID is known to be involved in extensive polar and hydrophobic interactions with the interior of the super-helix, specifically LRRs XIII-XXV [44]. In Arabidopsis, the ID is found between LRRs XXI and XXII. While this region is highly involved in hormone binding, the true hormone-binding site is an area between LRRs XXI and XXII or residues 559 to 743 [44]. The crystal structure of BRI1 shows the ID forming a groove that accommodates BR by destabilizing electrostatics [6]. Two loss of function alleles, *bri1-9* and *bri1-113* (S-662F and G-611E respectively), are located in this region [11,44,51]. It seems residues S-662 and G-611 are essential for LRR folding and are conserved in all sequences (Fig 5).

The binding of residues G-644, H-645, Y-646 and S-647 to a water molecule is also important in the protein folding and steroid binding [44,52,53]. All taxa except AtBRI1 had H-645 and S-647 replaced with Ser and Glu respectively, and TaBRI1 along with other monocot sequences had other polar residues substituted in this region. While it is known which residues are essential for binding water in this region in AtBRI1, the large number of hydrogen-bonding capable residues seen in TaBRI1 from residues 645 to 649 suggests BRI1 in monocots still uses a water molecule for steroid binding.

In contrast, hydrophobic regions play an essential role in the functional conformations of the BRI1 tertiary structure and provide a surface for the A and D rings of BL to communicate [6]. The alkyl chain of the steroid fits in a small pocket of the AtBRI1 protein composed of residues: I-563, W-564, M-657, F-658 [44]. While sequences differ in this binding pocket across taxa, hydrophobicity is mainly conserved, suggesting that function may not differ as drastically as residue identity may suggest (Fig 5). Despite monocot LRRs VI, X and XI being absent, the disulfide bridge between the cysteine residues of the ID and the LRRs is still likely to form as Cys is highly conserved.

### The juxtamembrane domain may vary in regulatory mechanism in activation of the KD

Many of these RLKs include dual-specificity protein kinases, such as BRI1, that phosphorylate both Ser/Thr and Tyr residues of target proteins. It seems that there are residues within the JTMD that are important for the KD activation [54]. The juxtamembrane residues Y-831, S-838, S-858, T-872 and T-880, which phosphorylate during activation of AtBRI1 [55], were conserved in all sequences (Fig 5). An investigation of the known *in vitro* phosphorylation sites found in the juxtamembrane domain revealed that the Thr residue T-846 was present only in AtBRI1. Likewise, T-842 was found in all sequences except GmBRI1 (Fig 5). I*n vivo* studies of AtBRI1 have suggested that both residues do not become phosphorylated [55]. It is also known that some of the Ser/Thr and Tyr residues, which may be phosphorylated, are not essential for kinase function. This may be explained by hierarchical nature of residue phosphorylation in AtBRI1 contributing to the absence of phosphotyrosine seen *in vitro*, as Tyr tends to be phosphorylated last [54].

The juxtamembrane domain is known to be important as a regulator, though its effect on kinase activity can vary. Interestingly, Y-831F in AtBRI1 shows a phenotype with larger leaves (with altered shape) whereas T-842A and S-858A show decreased phosphorylation, showing that residues within the juxtamembrane domain can be negative regulators of kinase function [14]. In addition, mutation of T-872A in Arabidopsis and *Solanum lycopersicum* increased kinase activity, demonstrating the juxtamembrane domain’s negative-regulatory role [38]. When these mutations were performed on the equivalent *in vitro S*. *lycopersicum* residues, peptide phosphorylation analysis showed the residues were positive regulators of kinase activity [38]. Regardless, primary sequence around T-842 seems to be a region where dicots and monocots differ as wheat and other monocot sequences lack AtBRI1 equivalent residues (Fig 5). This may suggest differences in phosphorylation mechanisms between AtBRI1 and other plant species studied in this investigation.

The KD in BRI1 is the catalytically essential portion of the protein. The activation loop, a region from residues 1038 to 1057 in AtBRI1 showed 100% conservation in all taxa.Y-956, known to be essential in catalytic function, was conserved in all taxa [14]. T-982, found in the KD and an essential residue for kinase function, was only found in AtBRI1 and SbBRI1 (Fig 5). Because most residues at this location were Ala or Ile, BRI1 of those taxa including TaBRI1, may have some variation in mechanism of kinase activity (Fig 5). It is not uncommon, however, for either Ser or Thr residues to be absent at the equivalent T-982 position in similar RLKs of multiple taxa. In a study conducted to analyze KDs of RLKs, only half of all RD RLKs, a class of plant RLKs characteristic of the Arg-Asp motif seen at residue 1008, had Ser or Thr at this position [55]. It is likely that this feature has a phylogenetic explanation as 5 out of the 6 monocot sequences had an Ala in place of the AtBRI1 T-982 equivalent (Fig 5).

The phosphorylation of S-1044, found in the activation loop is known to interact with a positively charged pocket consisting of: R-922, R-1008, D-1009, R-1032 [39]. As expected, S-1044 and other residues present in the positively charged pocket, R-922, R-1008, D-1009 and R-1032, were all conserved in wheat and other taxa studied (Fig 5). High conservation of the activation loop suggests that the dual specificity function of the TaBRI1 enzyme is intact.

### Monocot and dicot BRI1 show related but distinct evolutionary history

While a phylogenetic perspective is useful in gene family analyses, the unrooted phylogram is not a true representation of the historical relationships among the protein sequences. However, estimates based on percent identity can be drawn from the differences among taxa. The difference in the N_1_/ N_2_ ratio between monocots and dicots is reaffirmed by the separation of the two clades in the phylogram. It’s possible that variability in pre-ID LRRs can be due to gene duplication events or recombination of plant RLK orthologs [56]. This observation fits with the fact that Fabales (includes *G*. *max*) and Brassicales (includes *A*. *thaliana*) are two clades that rapidly diversified after they diverged ~100 MYA [57]. While this is sooner than the monocot/dicot divergence, differences between GmBRI1 and AtBRI1 can be explained by this independent evolutionary event.

Most of the differences in sequence are in the LRR region, so why then is the KD so well conserved? The enormous diversity of RLKs in plants had to have occurred as a result of signal transduction pathways becoming sensitive to previously unrecognized ligands. Receptor-like proteins (RLPs) lack kinase function and mainly interact with RLKs by forming complexes [58]. It is possible that proteins with novel functions can arise from the fusion of RLKs and RLPs. Thus, the intracellular signal transduction pathway relies on the same KD coupled with a new extracellular domain. The highly conserved KD is therefore a result of an essential function and likely shows very little change over time. New insight in the conservation and evolution of BRI1 and its interacting proteins will lead to a more complete picture of the functions involved in this complex gene.

## Supporting Information

S1 Fig Phylogenetic analysis of BRI1 in 23 dicot and 7 monocot plant species. The taxon identifier is shown followed by the gene ID. The monocots and dicot BRI1 proteins form two distinct clades.
(TIF)
