# Supplementary figures and images for: Evolutionary, Comparative and Functional Analyses of the Brassinosteroid Receptor Gene, BRI1, in Wheat and Its Relation to Other Plant Genomes
Source: PLoS One. 2015 May 28;10(5):e0127544. doi: 10.1371/journal.pone.0127544 (PMC4447442; doi:10.1371/journal.pone.0127544)

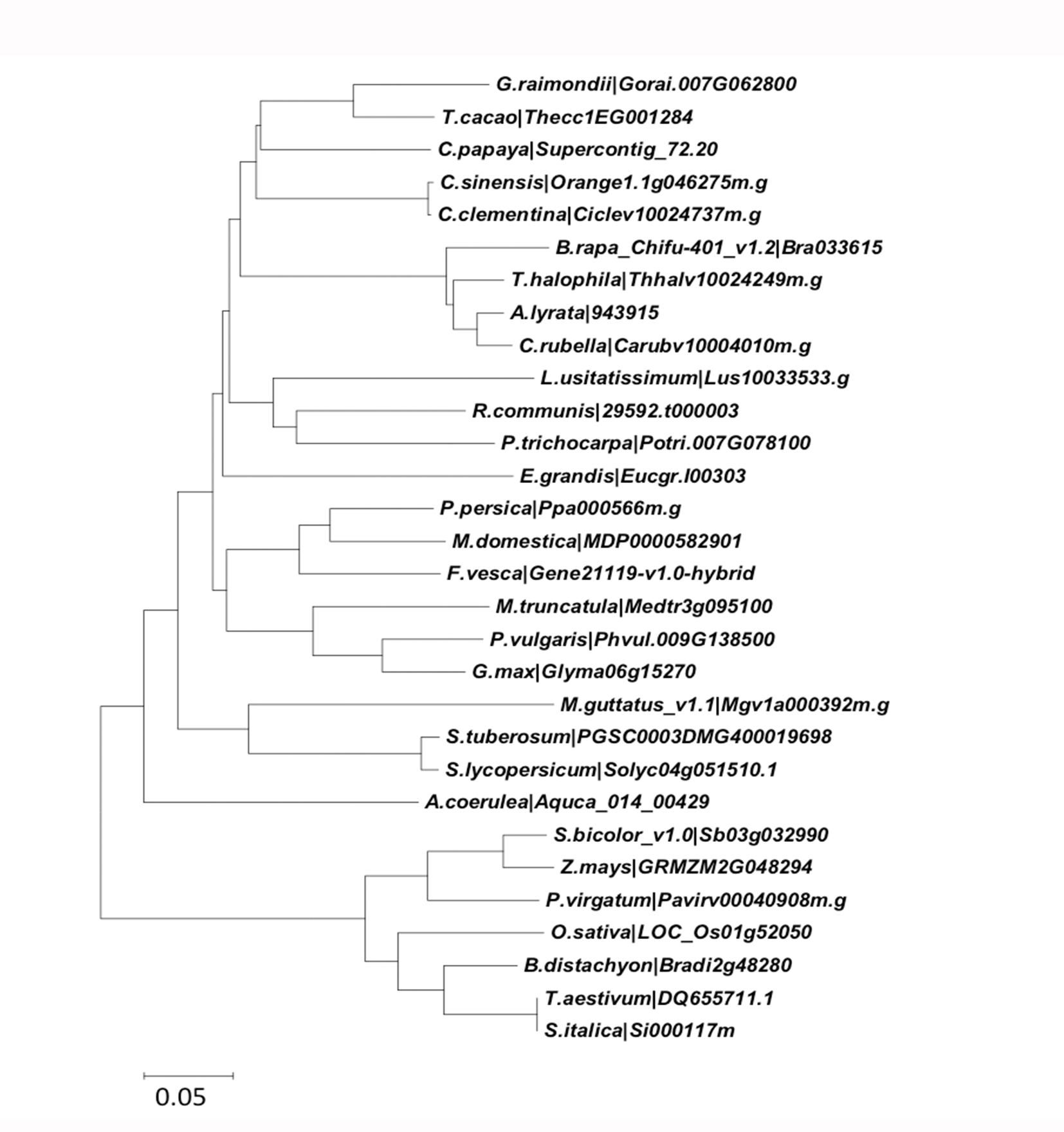

Supplement: S1 Fig — The taxon identifier is shown followed by the gene ID. The monocots and dicot BRI1 proteins form two distinct clades. (TIF) [file pone.0127544.s001.tif]
